# Supplementary material for: Asymmetric Synthesis of Quaternary Hydantoins via a Palladium-Catalyzed Aza-Heck Cyclization
Source: J Am Chem Soc. 2025 Nov 14;147(49):44692–8. doi: 10.1021/jacs.5c16022 (PMC12703750; doi:10.1021/jacs.5c16022)
Supplement: Supplementary file 2 [file ja5c16022_si_002.zip › All NMR FID Files/19/19_CarbonNMR/1/pdata/1/email_MLM03157B-1_1_1.pdf]

C13APT1024 CD3CN /opt/nmrdata/ mlmurr 2

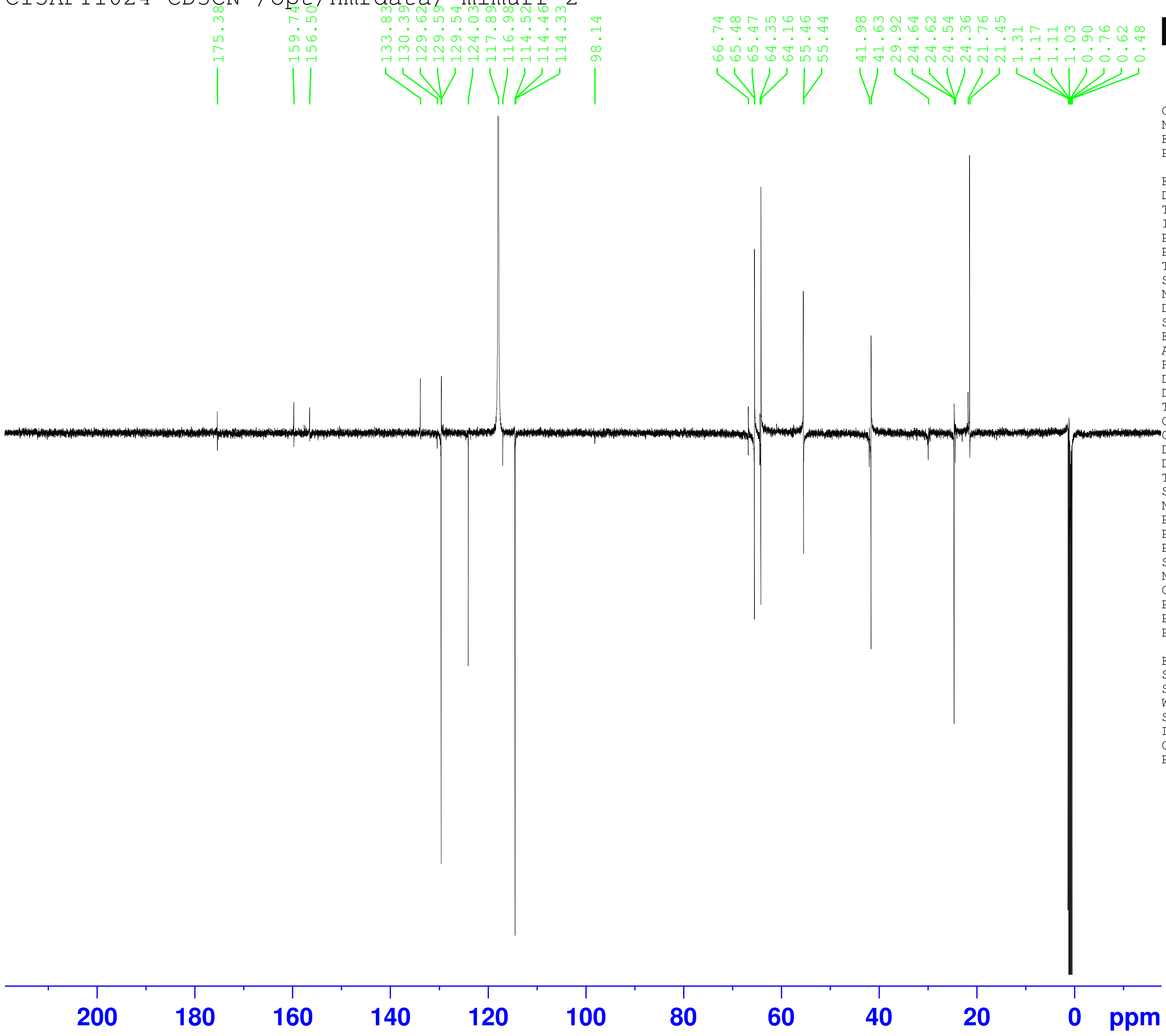

Current Data Parameters

NAME	MLM03157B-1
EXPNO	1
PROCNO	1

F2 - Acquisition Parameters

Date_	20250718
Time	20.15 h
INSTRUM	Avance Neo600 U of DE
PROBHD	Z168773_0053
PULPROG	jmod
TD	65536
SOLVENT	CD3CN
NS	2048
DS	4
SWH	35714.286 Hz
FIDRES	1.089913 Hz
AQ	0.9175040 sec
RG	101
DW	14.000 usec
DE	18.00 usec
TE	297.5 K
CNST2	145.0000000
CNST11	1.0000000
D1	2.00000000 sec
D20	0.00689655 sec
TD0	1
SFO1	150.9656791 MHz
NUC1	13C
P1	10.00 usec
P2	20.00 usec
PLW1	80.53800000 W
SFO2	600.3224013 MHz
NUC2	1H
CPDPRG2	waltz65
PCPD2	80.00 usec
PLW2	21.61000000 W
PLW12	0.42583000 W

F2 - Processing parameters

SI	32768
SF	150.9504981 MHz
WDW	EM
SSB	0
LB	1.00 Hz
GB	0
PC	1.40
